# Electrical lower esophageal sphincter augmentation in patients with GERD and severe ineffective esophageal motility—a safety and efficacy study

**DOI:** 10.1007/s00464-018-06649-y

**Published:** 2019-01-22

**Authors:** Matthias Paireder, Ivan Kristo, Reza Asari, Gerd Jomrich, Johanns Steindl, Erwin Rieder, Sebastian F. Schoppmann

**Affiliations:** Department of Surgery, Upper-GI-Service, Comprehensive Cancer Center GET-Unit, Medical University of Vienna, Spitalgasse 23, 1090 Vienna, Austria

**Keywords:** Electrical stimulation of lower esophageal sphincter, Postoperative dysphagia, Gastroesophageal reflux disease, Ineffective esophageal motility

## Abstract

**Background:**

Laparoscopic fundoplication (LF), even if performed in specialized centers, can be followed by long-term side effects such as dysphagia, gas bloating or inability to belch. Patients with an ineffective esophageal motility (IEM) and concurrent GERD are prone to postoperative dysphagia after LF. The aim of this study is to evaluate the safety and efficacy of electrical lower esophageal sphincter stimulation in patients with IEM and GERD.

**Methods:**

This is a prospective, open-label single center study. Patients with PPI-refractory GERD and ineffective esophageal motility were included for lower esophageal sphincter electrical stimulation (LES-EST). Patients underwent prospective follow-up including physical examination, interrogation of the device and were surveyed for changes in the health-related quality of life score.

**Results:**

According to power analysis, 17 patients were included in this study. Median distal contractile integral (DCI) was 64 mmHg s cm (quartiles 11.5–301). Median total % pH < 4 was 8.9 (quartiles 4–21.6). Twelve patients (70.6%) underwent additional hiatal repair. At 1-month follow-up, none of the patients showed any clinical or radiological signs of dysphagia. There were no procedure related severe adverse events. Mean total HQRL improved from baseline 37.53 (SD 15.07) to 10.93 (SD 9.18) at follow-up (FUP) (mean difference 24.0 CI 15.93–32.07) *p* < 0.001.

**Conclusions:**

LES-EST was introduced as a potential technique to avoid side effects of LF. LES-EST significantly improved health related quality of life and does not impair swallowing in patients with GERD and ineffective esophageal motility.

Gastroesophageal reflux disease (GERD) is a clinical condition characterized by reflux of gastroduodenal contents in to the esophagus with increasing prevalence over the last decades worldwide [1]. Although medical treatment with proton pump inhibitors (PPI) is effective, surgical treatment remains relevant in a large amount of patients refractory to PPI therapy [2]. Although laparoscopic fundoplication (LF) can be associated with side effects such as postoperative dysphagia, gas bloating or inability to belch, it is still considered the standard surgical procedure [3]. In particular, patients with ineffective esophageal motility (IEM) are prone to postoperative dysphagia after LF [4].

Recently, lower esophageal sphincter electrical stimulation (LES-EST) was introduced as an alternative surgical technique for the treatment of GERD in order to avoid side effects related to LF. It was demonstrated that LES-EST significantly increases LES pressure and improves typical GERD symptoms such as heartburn and regurgitation [5, 6].

The aim of this prospective study is to evaluate the safety and efficacy of LES-EST stimulation in patients with severe esophageal motility disorder requiring surgical GERD therapy [7].

## Materials and methods

### Study protocol

This is a prospective, open-label, non-randomized single-center study. Patients, assessed for anti-reflux surgery, received esophageal functional testing. Those who showed signs of IEM according to the Chicago classification v3.0 were prospectively screened for eligibility to undergo electrical LES-stimulation [7].

Primary endpoint of this study was safety assessment including medical morbidity associated with the device and/or the implantation procedure. Secondary endpoint was the clinical outcome, defined by improvement of GERD symptoms, measured with the GERD HRQL score, as well as the assessment of postoperative side effects such as dysphagia [8].

Inclusion criteria included indication for implantation of the LES-EST system, meeting the criteria of IEM and providing a signed informed consent. Exclusion criteria were met, if the patient was within a vulnerable population or was unable to understand the informed consent. Patients failing to attend follow-up visits were not eligible to participate at this study. Please find eligibility criteria in Table 1.

**Table 1:** Eligibility criteria

Inclusion criteria
Age between 18 and 80 years
History of GERD > 1 year
History of PPI usage and/or continued symptoms in spite of PPI usage
Ineffective esophageal motility according to the Chicago classification v3.0
Body mass index (BMI) < 35 m^2^/kg
Exclusion criteria
History of Barrett’s esophagus (> c1, > m1) or with any dysplasia
Type 1 diabetes or uncontrolled type 2 diabetes with HbA1c > 9.0
History of any esophageal or gastric malignancy
Persistent cardiac arrhythmia or cardiovascular disease
Presence of esophageal stenosis or stricture
Patients unwilling or unable to attend follow-up visits
Patient within a vulnerable population

Preoperative workup included upper-GI endoscopy as well as esophageal functional testing including high-resolution impedance manometry (InSIGHT Ultima®, Sandhill Scientific Inc., USA) and 24 h impedance/pH reflux monitoring (ZepHr®, Sandhill Scientific Inc., USA).

Short-term follow-up was performed 1 month after surgery. This consisted of physical examination, interrogation of the device and health-related quality of life (HRQL) assessment with standardized questionnaires (GERD-HRQL for heartburn and regurgitation) [8]. Dysphagia was graded from 0 to 4, according to the standardized classification used by Mellow and Pinkas [9].

### Implantation technique

**Fig. 1:**
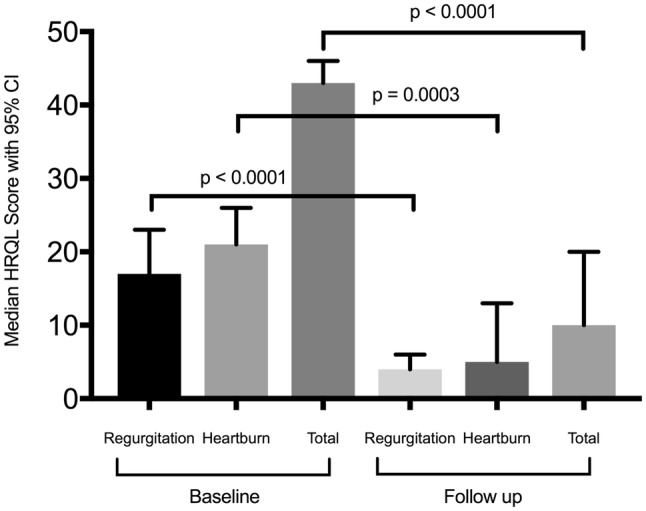
EndoStim Gen II device

**Fig. 2:**
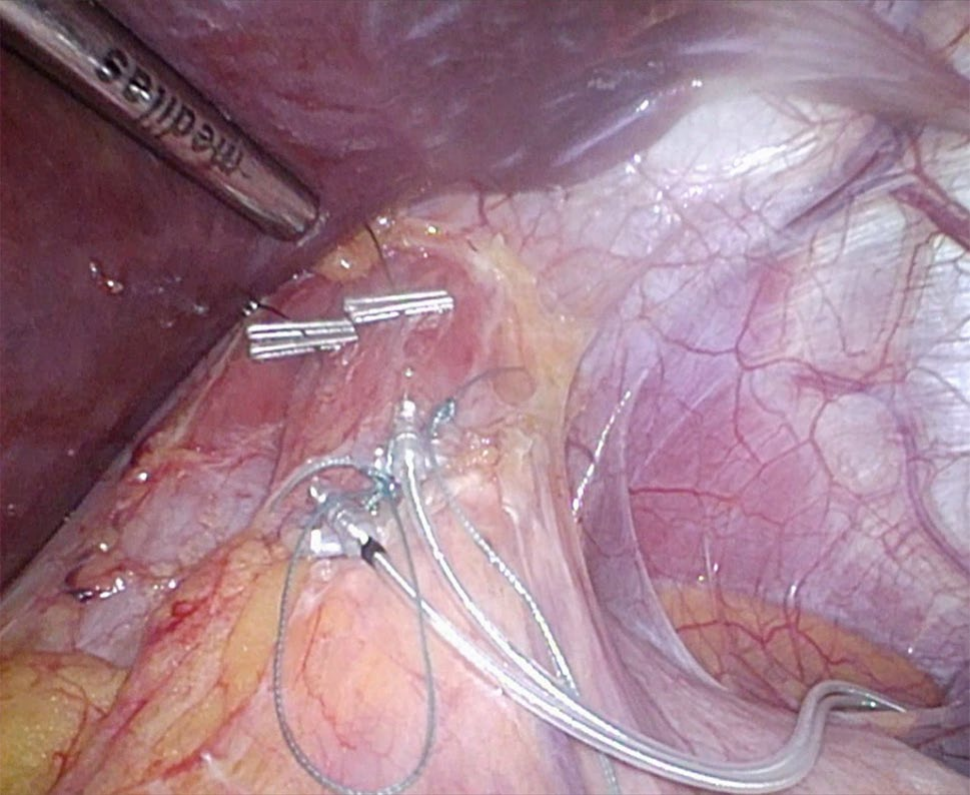
Intraoperative site after implanting the stitch electrodes

**Fig. 3:**
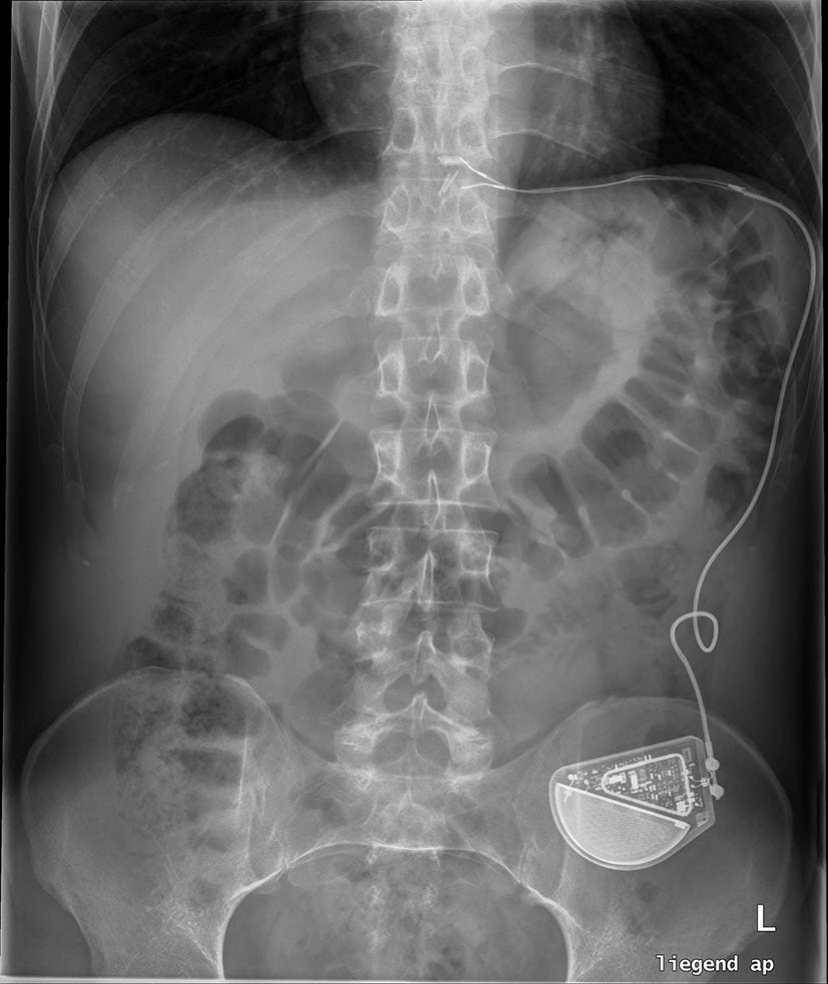
Abdominal X-ray showing the position of the electrodes and the implantable impulse generator (IPG)

Device-Implantation (Generation-II-LES-Stimulator Modell 1006 by EndoStim BV, the Hague, Netherlands) was performed as described previously [10] (Fig. 1). In brief, with the patient in anti-Trendelenburg position a minimal dissection of the abdominal and lower mediastinal esophagus was performed using an ultrasound based energy device. In the case of hiatal hernia, a complete mobilization of the distal esophagus and posterior hiatal repair was performed. The two stimulation electrodes were placed under endoscopic control at the anterior side of the EGJ approximately 1.5 cm apart and were fixed with 3/0 multifilament, non-absorbable thread, which is applied at least at one side of each silicone butterfly [11] (Fig. 2). A contrast swallow with Diatrizoate was performed at day 1 after surgery as well as an abdomen X-ray for documentation of the position of the lead and electrodes (Fig. 3). Patients were encouraged to take in soft diet for 4 days.

### Statistical analysis

SPSS (version 21.0, SPSS Inc., Chicago, IL, USA) was used for statistical analysis.

Sample size calculation was done in line with the original study protocol by Rodriguez et al. for mid- and long-term analysis of the efficacy of LES-EST. Sixteen patients are required at an alpha level of 0.05 and a beta level of 0.2 to detect a 10-point improvement in the GERD-HRQL score from baseline to the 6-month follow-up, assuming a standard deviation of 10 points [11].

All variables were depicted as median and interquartile range (IQR) or 95% confidence intervals (CIs) or mean with standard deviation (SD). Ineffective esophageal motility (IEM) was defined as a Distal Contractile Integral (DCI) below 450 mmHg s cm in ≥ 5 of out 10 swallows, according to the Chicago classification v3.0 [7]. Operating time was defined as the period between the placement of the last trocar and the removal of the first trocar. GERD health related quality of life (HRQL) scores before and after treatment were compared with paired *t* test due to normal distribution. *p* values < 0.05 were considered significant. Graphing was performed with GraphPad Prism (version 7.0c, GraphPad Software, Inc., La Jolla, CA, USA).

The study was approved by the ethics committee (EK 1217/2017) of the Medical University of Vienna.

## Results

### Patients

Seventeen patients (11 male, 64.7%) were included according to the study protocol. Mean age was 48.9 (SD 12.6) years. Mean body mass index (BMI) was 25.0 (SD 4.8) kg/m^2^. Eleven patients (64.7%) were on daily PPI at time of surgery. Eleven patients (64.7%) presented with typical and 9 patients (53.9%) with atypical GERD symptoms. The median GERD-HRQL score was 43 (quartiles 22–47). Preoperative esophageal functional testing found positive symptom correlations in all patients. Median total % of pH < 4 was 8.9 (quartiles 4–21.6). All patients fulfilled the criteria for IEM according to the Chicago classification version 3.0 (median DCI was 64 mmHg s cm, quartiles 11.5–301). For further baseline details, please see Table 2.

**Table 2:** Baseline characteristics and preoperative esophageal functional testing

Characteristic	*N* (%)	Mean (SD)°/median (IQR)*
Age, years*	17	48.9 (12.6)°
Body mass index (BMI)*	17	25.0 (4.8)°
Gender		
Male	11 (64.7)	
Female	6 (35.3)	
BMI class		
Normal (< 25)	11 (64.7)	
Overweight (≥ 25 and < 30)	4 (23.5)	
Obese (≥ 30)	2 (11.8)	
Patients using daily PPI	11 (64.7)	
Typical GERD symptoms	11 (64.7)	
Atypical GERD symptoms	9 (52.9)	
GERD-HRQL score		
Heartburn (IQR)		21 (15–27)*
Regurgitation (IQR)		17 (11–23.5)*
Total (IQR)		43 (22–47)*
Esophageal functional testing		
Total % pH time < 4		8.9 (4–21.6)*
Upright % pH time < 4		7.8 (1.5–20.3)*
Supine % pH time < 4		14.4 (1.7–20.7)*
Nr of reflux events		81 (52.3–100.8)*
LES resting pressure (mm HG)		15 (12.7–23.4)*
DCI mmHg s cm		64 (11.5–301)*

Values in parentheses are percentages unless indicated otherwise; values are mean (standard deviation, SD)

*PPI* proton pump inhibitors, *GERD* gastro esophageal reflux disease, *HRQL* health related quality of life, *LES* lower esophageal sphincter, *DCI* distal contractile integral

*Values are median (interquartile range, IQR)

### Surgery

All patients underwent laparoscopic implantation of the LES stimulation device. Twelve patients (70.6%) underwent additional hiatal repair. Median operating time was 45 min (quartiles 34–61). All patients were stimulated with 5 mA, 20 Hz and 220 µs pulse width. Median electrical impedance after implantation was 328.0 Ω(quartiles 301.75–366.0). In one patient, the placement of the electrodes had to be repeated due to stimulation failure (impedance was out of reach). There were no adverse events during surgery.

### Follow-up

Contrast swallows performed on postoperative day 1 did not show any leaks, obstructions or surgical complications. In patients, who underwent hiatal hernia repair, no recurrence was detected. There was no dysphagia reported, neither during the immediate postoperative course nor at the follow up visit 1 month later. Six patients (35%) still took PPI 1 month after surgery. There were no reports of gastrointestinal side effects such as bloating or inability to belch (Table 3).

**Table 3:** Postoperative adverse events

Event*	*N* events
Dysphagia	0
Inability to belch	0
Bloating	0
Abdomen/chest pain	0
Nausea/vomiting	0
Pain at implant site°	2 (11.7%)

*Events were routinely screened according to prospective study protocol on postoperative day 1 and at follow-up

°Pain at implant site resolved within days without treatment

**Fig. 4:**
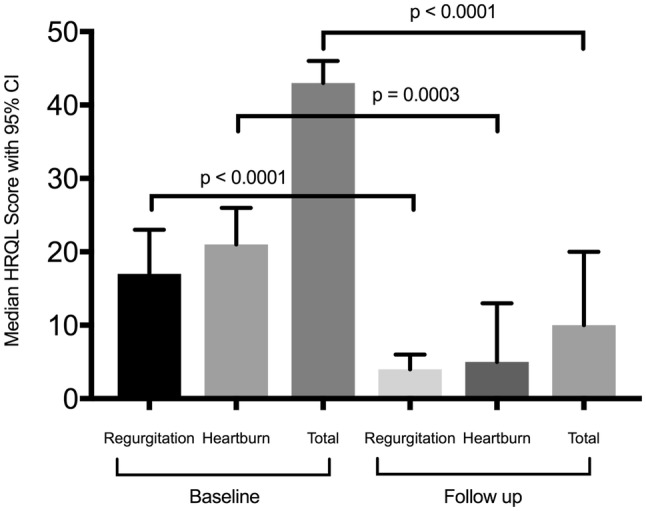
Improvement in GERD symptoms measured by the health-related quality of life (HRQL) score. Columns are median with 95% CI. Differences are compared using the paired *t* test

Mean total HQRL improved from baseline 37.53 (SD 15.07) to 10.93 (SD 9.18) at follow up (FUP) (mean difference 24.0 CI 15.93–32.07) *p* < 0.001. Mean HRQL for heartburn also improved from baseline 20.53 (SD 7.80) to 7.0 (SD 7.08) at FUP (mean difference 12.0 CI 9.61–17.32) *p* < 0.001. Also the HRQL score for regurgitation improved significantly from baseline 17.0 (SD 8.59) to FUP 4.2 (SD 4.48) (mean difference 11.73 CI 7.25–16.22) *p* < 0.001 (Fig. 4). Electrical impedance rose from mean 351.33 (SD 72.66) Ohm at implantation to 403.55 (SD 92.2) at FUP, *p* = 0.019. No changes were made to the stimulation protocol at the follow-up.

## Discussion

This is the first prospective study investigating the safety and efficacy of electrical lower esophageal sphincter stimulation in patients with GERD and IEM. The data presented provide evidence that LES-EST is safe and does not impact postoperative swallowing in this special subgroup of surgical GERD patients and is effective 1 month after surgery.

Ineffective esophageal motility is frequently seen in patients with GERD [12]. However, the association between GERD and IEM is yet not fully understood and discussed very controversially [13, 14]. Notably, there are numerous reports, which show a possible connection between the IEM and impaired mucosal integrity caused by GERD [12, 15, 16].

With the introduction of the high resolution manometry, the diagnosis of IEM has been updated [17]. Definition of IEM was changed from the conventional manometry, contractions exhibiting amplitudes < 30 mmHg, to the use of the DCI, not exceeding 450 mmHg s cm in ≥ 50% of the swallows [7]. This might jeopardize comparability to earlier studies dealing with anti-reflux surgery in patients with esophageal dysmotility. To our knowledge, this is the first prospective trial investigating anti-reflux surgery in patients with IEM using the updated Chicago classification.

Novitzky et al. published a retrospective analysis of patients with severe dysmotility undergoing LF [4]. Although these were not IEM patients, according to the actual classification, an early dysphagia rate of 73% was reported, which required several interventions. Finally, only in 4.2% of the cases dysphagia remained persistent. Postoperative dysphagia severely impacts patient’s well-being. It is only recently that Kapadia et al. published the relationship between HRM findings and postoperative dysphagia. Although patients did not meet the Chicago criteria v3.0 of IEM in this series, they could show a significant correlation between preoperative DCI and post-fundoplication dysphagia [18].

LES-EST was introduced by Rodriguez et al. in 2012 showing that LES pressure could be increased significantly by controlled electric stimulation, without causing any complaint of dysphagia [5]. It is of significant advantage that LES stimulation does not have any effect on the LES relaxation or esophageal body function [19].

The presence of dysphagia after LES-EST has been described differently depending on simultaneous hiatal repair [10, 11]. The primary open-label trial did not show any signs of dysphagia [11]. Remarkably, no patients underwent hiatal repair due to strict inclusion criteria. However, in the international multicenter trial patients with small and medium sized hiatal hernia were included [10]. For the first time, a mild dysphagia rate was reported in the interim results. Four out of 42 patients mentioned mild to moderate dysphagia. All 4 patients underwent hiatal repair as well and dysphagia resolved without intervention.

In our study, the majority of patients underwent hiatal repair (70.6%). Yet, the crural repair before electrode implantation did not have any impact on postoperative dysphagia.

However, hiatal repair on its own might have an anti-reflux effect. There is an ongoing discussion about the effect of hiatal repair in anti-reflux surgery. A recently published comparative cohort study showed no improvement in patients who underwent hiatal repair without LNF or EGJ augmentation. New-onset abnormal acid exposure after surgery was seen in 38.9% of patients [20]. Although hiatal repair plays a significant role in anti-reflux surgery, this study found no satisfying primary effect. However, due to our low treatment number we cannot assess the role of hiatal repair in our collective.

This study has some limitations that need to be addressed. With regard to patient satisfaction and quality of life scores, there might be a placebo effect 1 month after surgery. This study, however, was designed to investigate dysphagia and GI side effects in a complex patient group. Those findings are much less influenced by a placebo effect. Taken into account the limited patient number, our results cannot be easily generalized.

In our personal experience, anti-reflux surgery in this patient group can be quite challenging. This newly established operation technique might have a place in foregut surgery. This enables surgeons to offer a personalized therapy option and can therefore reduce adverse effects. Despite the positive findings regarding GI symptoms after surgery, reflux control remains the key goal. Long-term monitoring of symptoms and objective pH measurements should be the subsequent step to endorse these findings.

## Conclusion

In conclusion, LES-EST is safe and has a favorable risk profile in patients with GERD and IEM. In a short term, there were no cases of dysphagia or other GI symptoms after electrical stimulation of the lower esophageal sphincter for GERD in patients with esophageal dysmotility.

## References

[1] El-Serag HB, Sweet S, Winchester CC, Dent J (2014). Update on the epidemiology of gastro-oesophageal reflux disease: a systematic review. Gut.

[2] Subramanian CR, Triadafilopoulos G (2015). Refractory gastroesophageal reflux disease. Gastroenterol Rep (Oxf).

[3] Richter JE (2013). Gastroesophageal reflux disease treatment: side effects and complications of fundoplication. Clin Gastroenterol Hepatol.

[4] Novitsky YW, Wong J, Kercher KW, Litwin DE, Swanstrom LL, Heniford BT (2007). Severely disordered esophageal peristalsis is not a contraindication to laparoscopic Nissen fundoplication. Surg Endosc.

[5] Rodriguez L, Rodriguez P, Neto MG, Ayala JC, Saba J, Berel D, Conklin J, Soffer E (2012). Short-term electrical stimulation of the lower esophageal sphincter increases sphincter pressure in patients with gastroesophageal reflux disease. Neurogastroenterol Motil.

[6] Rodriguez L, Rodriguez PA, Gomez B, Netto MG, Crowell MD, Soffer E (2016). Electrical stimulation therapy of the lower esophageal sphincter is successful in treating GERD: long-term 3-year results. Surg Endosc.

[7] Kahrilas PJ, Bredenoord AJ, Fox M, Gyawali CP, Roman S, Smout AJ, Pandolfino JE, International High Resolution Manometry Working G (2015). The Chicago Classification of esophageal motility disorders, v3.0. Neurogastroenterol Motil.

[8] Velanovich V (1998). Comparison of generic (SF-36) vs. disease-specific (GERD-HRQL) quality-of-life scales for gastroesophageal reflux disease. J Gastrointest Surg.

[9] Mellow MH, Pinkas H (1984). Endoscopic therapy for esophageal carcinoma with Nd:YAG laser: prospective evaluation of efficacy, complications, and survival. Gastrointest Endosc.

[10] Kappelle WF, Bredenoord AJ, Conchillo JM, Ruurda JP, Bouvy ND, van Berge Henegouwen MI, Chiu PW, Booth M, Hani A, Reddy DN, Bogte A, Smout AJ, Wu JC, Escalona A, Valdovinos MA, Torres-Villalobos G, Siersema PD (2015). Electrical stimulation therapy of the lower oesophageal sphincter for refractory gastro-oesophageal reflux disease—interim results of an international multicentre trial. Aliment Pharmacol Ther.

[11] Rodriguez L, Rodriguez P, Gomez B, Ayala JC, Saba J, Perez-Castilla A, Galvao Neto M, Crowell MD (2013). Electrical stimulation therapy of the lower esophageal sphincter is successful in treating GERD: final results of open-label prospective trial. Surg Endosc.

[12] Falcao A, Nasi A, Brandao J, Sallum R, Cecconello I (2013). What is the real impairment on esophageal motility in patients with gastroesophageal reflux disease?. Arq Gastroenterol.

[13] Kasamatsu S, Matsumura T, Ohta Y, Hamanaka S, Ishigami H, Taida T, Okimoto K, Saito K, Maruoka D, Nakagawa T, Katsuno T, Fujie M, Kikuchi A, Arai M (2017). The effect of ineffective esophageal motility on gastroesophageal reflux disease. Digestion.

[14] Felix VN, DeVault K, Penagini R, Elvevi A, Swanstrom L, Wassenaar E, Crespin OM, Pellegrini CA, Wong R (2013). Causes and treatments of achalasia, and primary disorders of the esophageal body. Ann N Y Acad Sci.

[15] Chrysos E, Prokopakis G, Athanasakis E, Pechlivanides G, Tsiaoussis J, Mantides A, Xynos E (2003). Factors affecting esophageal motility in gastroesophageal reflux disease. Arch Surg.

[16] Fuchs HF, Gutschow CA, Brinkmann S, Herbold T, Bludau M, Schroder W, Bollschweiler E, Holscher AH, Leers JM (2014). Effect of laparoscopic antireflux surgery on esophageal motility. Dig Surg.

[17] Pandolfino JE, Fox MR, Bredenoord AJ, Kahrilas PJ (2009). High-resolution manometry in clinical practice: utilizing pressure topography to classify oesophageal motility abnormalities. Neurogastroenterol Motil.

[18] Kapadia S, Osler T, Lee A, Borrazzo E (2017). The role of preoperative high resolution manometry in predicting dysphagia after laparoscopic Nissen fundoplication. Surg Endosc.

[19] Eypasch E (2014). Electrical stimulation of the lower oesophageal sphincter: an emerging therapy for treatment of GORD. Eur Surg.

[20] Furnee EJ, Draaisma WA, Gooszen HG, Hazebroek EJ, Smout AJ, Broeders IA (2011). Tailored or routine addition of an antireflux fundoplication in laparoscopic large hiatal hernia repair: a comparative cohort study. World J Surg.

